# Indexing hematological and serum biochemical reference intervals of Himalayan snow trout, *Schizothorax esocinus* to instrument in health assessment

**DOI:** 10.3389/fphys.2023.989442

**Published:** 2023-03-24

**Authors:** Quseen Mushtaq Reshi, Imtiaz Ahmed, Khalid Mashay Al-Anazi, Mohammad Abul Farah

**Affiliations:** ^1^ Fish Nutrition Research Laboratory, Department of Zoology, University of Kashmir, Srinagar, India; ^2^ Department of Zoology, College of Science, King Saud University, Riyadh, Saudi Arabia

**Keywords:** fish, reference intervals, hematology, serum biochemistry, *Schizothorax*

## Abstract

*Schizothorax esocinus*, commonly known as snow trout, is one of the main contributors of food and livelihood in the colder zone of Himalayan region. The comprehensive information on its hematological and serum biochemical reference intervals is not reported yet. In the present study an attempt has been made to elucidate the hematological and serum biochemical reference intervals of *S. esocinus* from River Jhelum using protocols of the American Society of Veterinary Clinical Pathology (ASVCP). Wild fish were sampled over a period of 2 years from the pollution free sites of river Jhelum. Fish blood was harvested through caudal venipuncture and hemato-biochemical analysis performed thereof. Data values from a total of healthy 432 adult fish specimens (216 male, 216 female) were systematically recorded. The reference intervals for hematological and serum biochemical parameters of *S. esocinus* were established using Reference Value Advisor software v 2.1. RIs for hematological and serum analytes ranged as: hemoglobin (Hb) 78.38–116.35 (g/L); white blood cells (WBC) 10–20 (×10^9^/L); red blood cells (RBC) 1.30–2.15 (×10^12^/L); packed cell volume 27.00–39.45 (%); total protein 39.21–61.62 (g/L); albumin 8.20–22.02 (g/L); globulin 27.58–49.55 (g/L); glucose 3.25–7.18 (mmol/L); urea 0.96—2.38 (mmol/L); cholesterol 3.80–6.90 (mmol/L). The study also depicted that certain blood measurands were influenced with respect to sex. Significantly (*p <* 0.05) higher values of Hb, red blood cells count and serum glucose were noted in male as compared to female which, on the other hand, registered higher white blood cells count and serum cholesterol level (Mann Whitney *U* test, *p <* 0.05). The work, therefore, provides baseline information on hematological and serum biochemical analytes of this species which holds high commercial importance. RIs reported here can help monitor the health status of fish by improving the use of non-lethal diagnostic methods in piscine medicine.

## Introduction

Blood is a vital fluid consisting of a variety of cells in the plasma medium. Analysis of blood is a common tool to evaluate physiological status of an organism including fish. Recent research shows that hematological parameters are important indicators of fish health status and are used in innovative methods (Fazio, 2019; Fazio et al., 2019). Among all the diagnostic tools used to evaluate the health of organism including fish, hematological and serum biochemical assessments are recognized as easy and reliable methods for analyzing the health status of aquatic animals as these indices provide speedy and reliable information on metabolic disorders, diseases, deficiencies and chronic stress status (Ahmed et al., 2020; Zakes et al., 2016). A multitude of endogenous and exogenous factors such as age, sex, season, breeding cycle, biological behavior, environment, health status, habitat as well as external factors like water temperature, environmental quality, seasonal dynamics, food and stress influence the hematological parameters of fish while the quantitative determinations of various serum analytes facilitate in assessment of the functional status of the vital body organs like the kidney, liver, heart, pancreas, etc., and can predict the degree of organ damage as well (Ahmed et al., 2020; Ahmad et al., 2021a; Fawole et al., 2023; Khan and Khan, 2021; Reshi and Ahmed, 2022; Seibel et al., 2021). Although, blood is considered as an immediate reflector of health condition of fish but still hematological as well as serum data in piscine species are not optimally utilized due to the lack of reference ranges and appropriate interpretive skills (Nabi et al., 2022).

One of the intricacies in assessment of the state of health of natural fish populations has been the paucity of reliable reference ranges in the normal condition. In contrast to fish, where it is mainly understudied, this field has widely been expanded in veterinary medicine (Reshma et al., 2020). Development of database of normal hemato-biochemical ranges in fishes is of critical importance for assessment of health and successful running of aquaculture systems as well as maintaining the natural stocks of the fish (Reshi and Ahmed, 2022). Apart from hematological parameters, serum biochemical values act as reflectors of internal milieu of the fish and also help in detection and diagnosis of metabolic disturbances and diseases in fishes (Ferrari et al., 2007). Total protein, albumin and globulin levels in fish blood are used as basic indices of health status and condition (Ahmad et al., 2021a; Svoboda et al., 2001). These parameters along with other analytes are being used consistently in healthcare of humans and domestic animals as well.

Among all the aquatic ecosystems of Kashmir, river Jhelum, locally known as ‘Vyeth’ is the chief riverine system of the Himalayan region. It is one of the largest fisheries resources of the region and it supports a wide variety of fish fauna (Ahmad et al., 2021b; Reshi and Ahmed, 2020). It caters to considerable percentage of the fish demand of the human population. Of the various fishes found in the river, Cyprinids are predominant with main representatives from family Cyprinidae which include Schizothoracines*.* Among different *Schizothorax* species, *Schizothorax esocinus* (Heckel, 1838) is commonly called as snow trout and locally known as ‘Chirruh’. It is one of the endemic and economically important cold water food fishes of Indian Himalayan colder zone in general and Kashmir region in particular (Reshi and Ahmed, 2020). The fish species found in most of Asian countries like India, Pakistan, China, Afghanistan and Nepal (Mir et al., 2014). It fetches high commercial price due to its gratifying taste and high food value (Ahmed, 2018). The work on aspects of *S. esocinus* has been reported in the past but these are mainly focused on its length-weight relationship, fecundity, growth, genome organization, however, the normal hematological and serum biochemical reference values of *S. esocinus* are not reported from any part of the world yet. Despite having high commercial importance, the fish is available only in the wild and its culture in the farms is not developed up to the mark yet. Therefore, the current study was undertaken to define the presently lacking RIs for hematological and serum biochemical parameters of *S. esocinus* using ASVCP protocol (Friedrichs et al., 2012). The study draws significance in terms of the lack of hematological data of this particular fish species which needs to be established. This has relevance in improving the applications of clinical diagnosis for benefitting the currently lacking culture of *Schizothorax* spp. Due to anthropogenic activities, the population of *Schizothorax* species are declining day-by-day and in order to start proper conservation and management of these important food fish species, monitoring of health status of the species is imperative which warrant the conscious study for establishment of reference values of the species, which could be instrumental for the assessment of health of these species both in natural and culture condition.

## Materials and methods

### Study sites and fish collection

**FIGURE 1:**
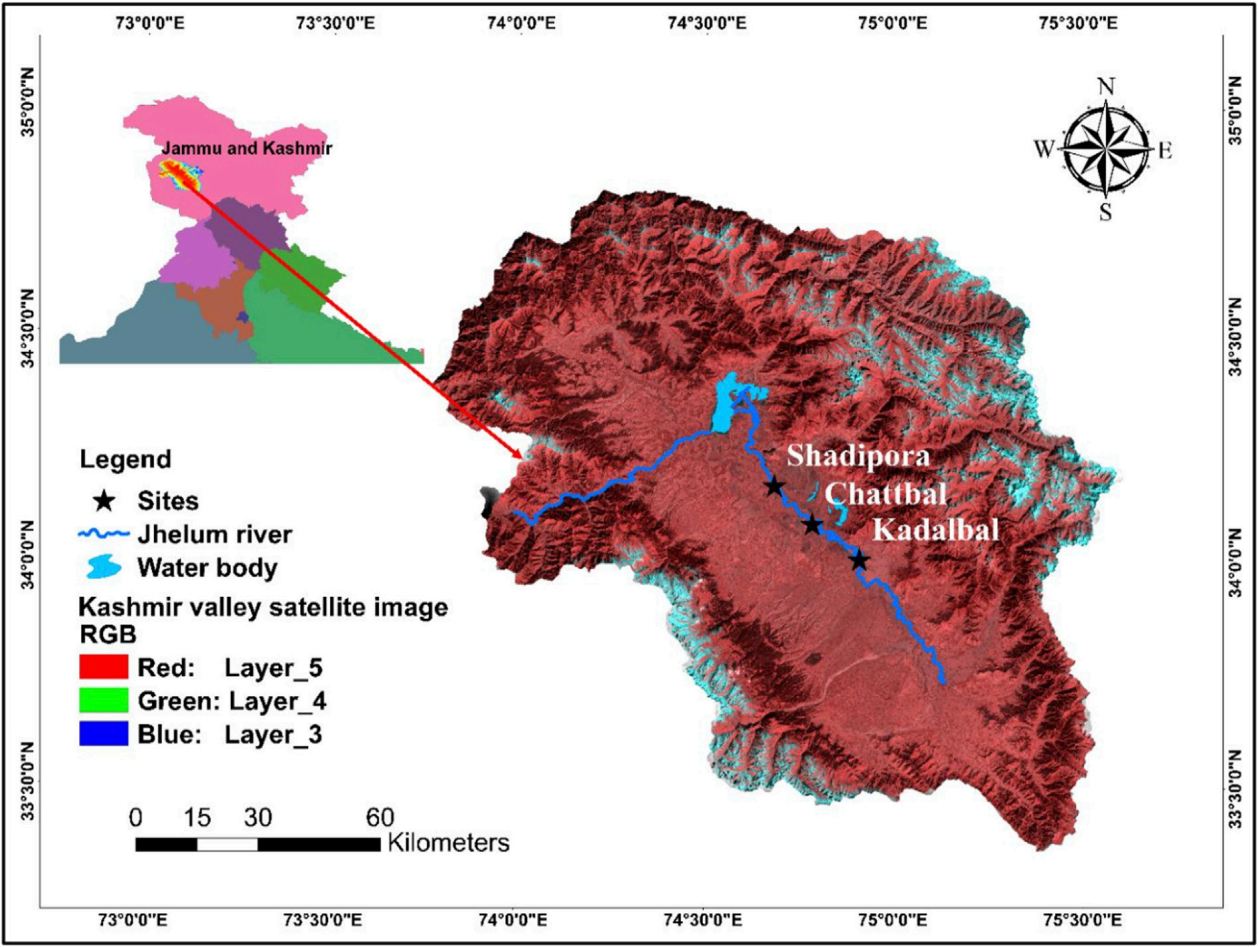
Map showing study sites of River Jhelum.

For the establishment of hematological and serum biochemical reference values, live adult specimens of *S. esocinus* (Average size = 33.47 ± 2.41 cm; Average weight = 400.68 ± 90.54 g) were captured by using cast nets with the help of local fishermen from three sites, i.e., Kadlabal (34°00′39″N, 74°54′38.43″E), Chattbal (34°05′23.89″N, 74°47′06.87″E) and Shadipora (34°10′26.85″N, 74°41′05.02″E) of River Jhelum, during the study period April 2017- March 2019. Length of river Jhelum in Kashmir valley is 129 km. The map showing study sites is given in Figure 1. Distance between site one and site two was nearly 15 km and the distance between site two and site three was nearly 13 km. Site selection was based on thorough survey of the study area by considering the various ecological (physico-chemical characteristic features including occurrence of contaminants or pollutants), biological [(availability of fishes, availability of food for fishes, quality and density of plankton) and micro-biological] factors (species and quantity of parasites). Fish were examined for gross morphology and necropsy, those with signs of injury, lesions and abnormal behaviour were eliminated from the study. All the fish specimens were adult and of nearly similar sizes. Sample collection was done on monthly basis for 2 years. Data values from a total of healthy 432 adult fish specimens (216 male, 216 females) were recorded. Sampled fishes were immediately transferred in plastic containers containing water and transported to wet laboratory and stocked in plastic tanks having continuous flow through water system facility for overnight acclimation of fishes. The fish were maintained in natural 12:12 h light dark photoperiod at a density of three fish per 70 L tank. The sex of the fish was identified by macroscopic examination of gonads. Besides, the morphological features such as body shape, size, anal fin length, dorsal fin spines were also observed to determine sex (Rai et al., 2002; Sharma and Mehta, 2010).

### Physico-chemical analysis

Water samples from the three study sites were collected each month of the 2-year study and assessed for physico-chemical parameters. i.e., temperature was recorded using mercury thermometer on spot; pH was recorded using digital pH meter (pHep-HI 98107, United States of America) on spot. The pH meter was standardized using suitable buffer solutions; dissolved oxygen was estimated manually by Winklerʼs method, fixed on the spot and estimated in laboratory; free carbon dioxide was estimated manually by titrimetric method using sodium hydroxide as titrant and phenolphthalein as an indicator in the laboratory; total alkalinity was estimated manually by titrimetric method using sulphuric acid as titrant, phenolphthalein and methyl orange as indicators in the laboratory (APHA, 1998).

### Hematological and serum biochemical analysis

In the laboratory, the fish were maintained for 1 day under normal photoperiod (Zhao et al., 2018). Blood was drawn from the caudal vein of the live fish using a sterile syringe and needle after using anesthesia (MS-222 at 0.3 gL^−1^). Acquisition of blood samples was quickly followed by immediate hematological and serum biochemical analysis. 2–3 mL blood sample was taken in heparin (anticoagulant) coated vials (AcCuvet-PLUS; Lithium Heparin; 25 I.U mL^-1^) and immediately used for hematological analysis. 2 mL of the blood directly from the syringe was immediately (within 1 min) taken in Eppendorf tubes, centrifuged at 5000 g for 5 min in centrifuge (Tarsons, Spinwin, MC-02) to obtain serum for biochemical analysis. Hemoglobin (Hb) was estimated by mixing 20 µL of blood in 5 mL of Drabkins reagent and left to stand for 15 min, then concentration was measured spectrophotometrically at 540 nm (Thermoscientific- Genesys 10 S UV-VIS) (Drabkin, 1946). The white blood cells (WBCs) and red blood cells (RBCs) were counted manually using Natt and Herrick’s diluent (1:200) and a Neubauer-ruled hemocytometer (Marienfeld-Superior, Lauda-Konigshofen, Germany) (Khan and Maqbool, 2017). The microhematocrit technique was used to determine packed cell volume (PCV) values after the capillary tubes containing blood had been spun in centrifuge (REMI RM-12C India) at 15300 g for 5 min and expressed as percentage (Khan and Maqbool, 2017). Erythrocyte sedimentation rate (ESR) was recorded by following Wintrobe Tube method wherein the anticoagulated blood was filled in a Wintrobe tube upto zero mark on top and kept undisturbed in vertical position in a rack. This allows the sedimentation of erythrocytes. After 1 h level of fall of the column of sediment was noted as ESR and expressed in mm per hour (Wedemeyer et al., 1983). These data were used to calculate the following erythrocyte indices: mean corpuscular hemoglobin (MCH), mean corpuscular hemoglobin concentration (MCHC) and mean corpuscular volume (MCV) according to Dacie and Lewis (1991). The serum was immediately analyzed in Biochemistry Auto Analyzer (Robert Riele GmbH and Co. KG, Germany) using standard commercial kits -(Erba Diagnostic Kits, Transasia Bio-medicals Ltd., Solan; In technical collaboration with: ERBA diagnostics Mannheim GmbH, Germany) wherein the total protein estimation was based on Biuret method; Albumin estimation was based on BCG (bromocresol green) Dye Method; The concentration of globulins was calculated (total protein minus albumin concentrations); Serum glucose estimation was based on GOD-POD (glucose oxidase-peroxidase) method; serum urea estimation was based on glutamate-urease method; cholesterol concentration was based by CHOD-PAP (cholesterol oxidase phenol 4-aminoantipyrine peroxidase) method (Ratnakar, 2016). Analysis was carried by the authors after proper guidance from the experts/service providers.

### Statistical analysis

Reference intervals for hematological and serum biochemical analytes were determined according to the published guidelines of American Society for Veterinary Clinical Pathology (ASVCP) which follow recommendations of Clinical Laboratory and Standards Institute (CLSI) (Friedrichs et al., 2012). Normality was assessed for each measurand by the Anderson-Darling test, where the *p*-value threshold limit of 0.05 was used. Mann Whitney *U* test was used to compare male *versus* female blood results, where the *p*-value threshold limit was 0.05. Data was analyzed statistically using Reference value Advisor software V 2.1 (Geffre et al., 2011). Data was screened for outliers using Tukeys/Dixon Reed outlier test by the software. After eliminating the outliers, the data was reanalyzed. Seasonal variations in hematological and serum biochemical analytes were assessed by one way ANOVA using SPSS software.

## Results

### Physico-chemical parameters

In laboratory, the average values of physico-chemical parameters were noted as temperature = 14.03°C ± 4.95 °C; pH = 8.11 ± 0.34; dissolved oxygen = 6.83 ± 1.17 mg/L; free carbon dioxide = 5.15 ± 2.42 mg/L; total alkalinity = 135.79 ± 26.64 mg/L. The results of physico-chemical parameters of water samples are summarized in Table 1. There was no significant variation in the parameters of river Jhelum and laboratory.

**TABLE 1:** Physico-chemical parameters of River Jhelum.

Physico-chemical parameter	Mean	Median	SD	Minimum	Maximum
Water temperature (°C)	12.94	13	5.04	4	21
pH	8.06	8.1	0.20	7.7	8.4
Dissolved oxygen (mg/L)	7.27	7.58	1.15	4.92	9.08
Free Carbon dioxide (mg/L)	5.02	4.4	2.40	1.76	10.12
Total alkalinity (mg/L)	150.38	150	27.51	88	201

### Hematological and serum biochemical reference intervals

**FIGURE 2:**
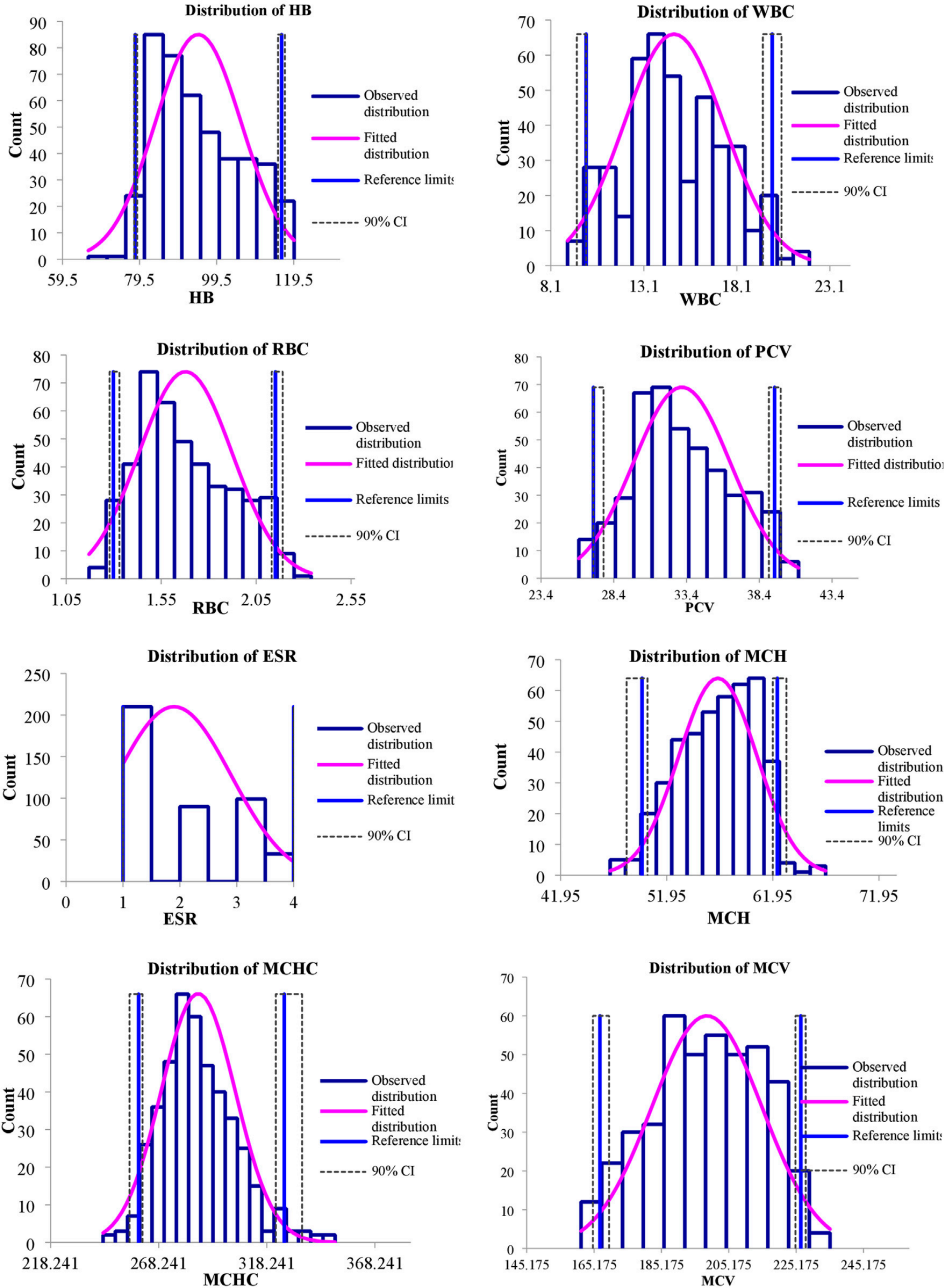
Representing distribution of hematological reference intervals of *S. esocinus* from River Jhelum.

**FIGURE 3:**
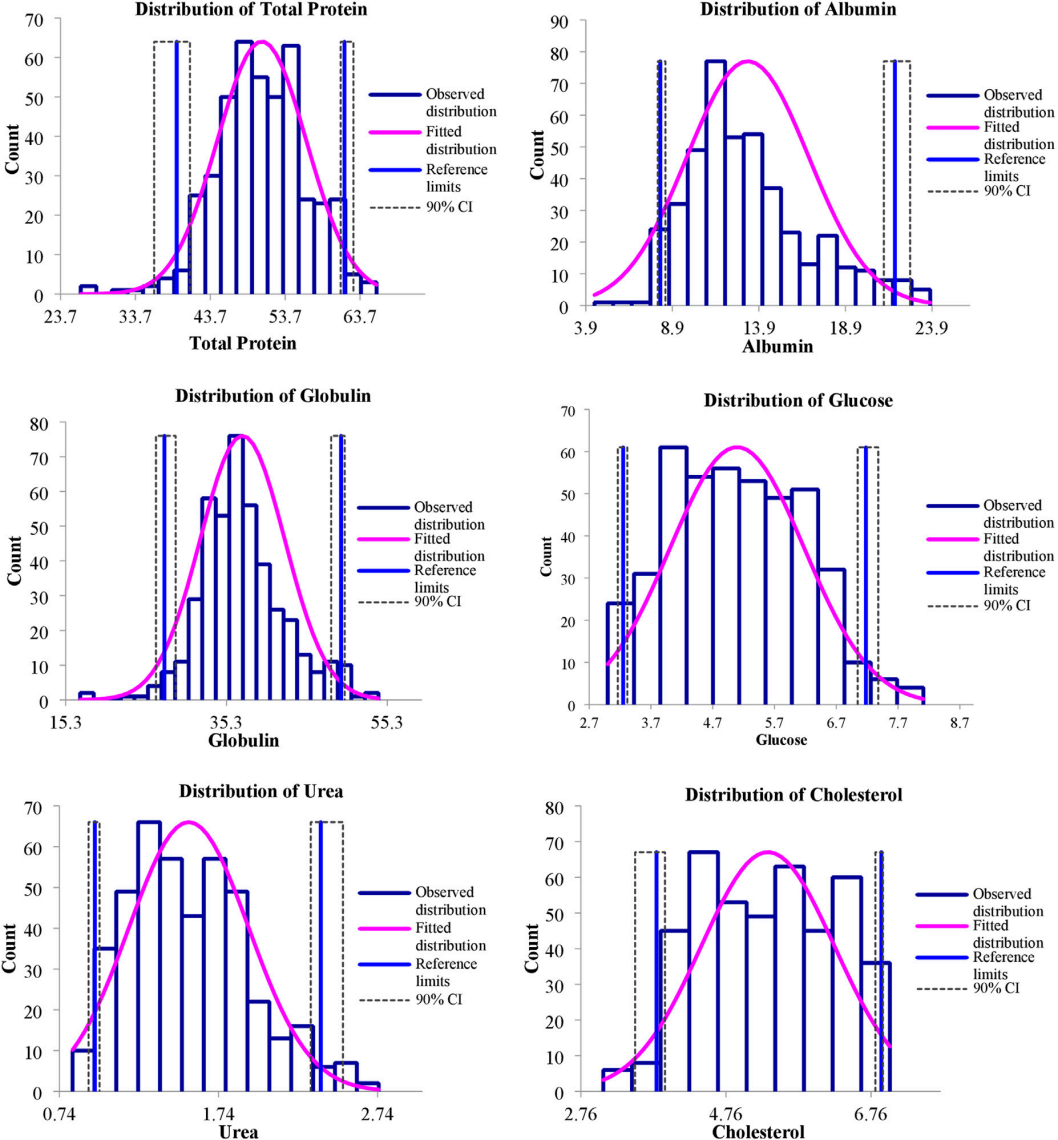
Representing distribution of serum biochemical reference intervals of *S. esocinus* from River Jhelum.

The values of mean, SD, coefficient of variation, minimum, maximum and reference intervals (90% Confidence Interval) for hematological and serum biochemical parameters of *S. esocinus* (combined sexes) are presented in Table 2. Among all the measured hematological variables, Hb and RBC were found to be significantly (*p* < 0.05) higher in male fish in comparison to female fish while WBC count was noted significantly (*p* < 0.05) higher in female fish as compared to the male fish (Table 3). In case of serum parameters, glucose was observed significantly (*p* < 0.05) higher in male fish and cholesterol values were significantly (*p* < 0.05) higher in female fish as presented in Table 3. Distribution curve of hematological and serum biochemical reference values of *S. esocinus* are given in Figures 2, 3. Overall, statistical analysis depicted significant (*p <* 0.05) differences in some blood analytes between the sexes. Our findings show that the values of Hb, RBC and glucose in male fish were significantly (*p <* 0.05) higher than that of female fish. Female fish showed higher values of WBC and serum cholesterol. Correlation matrix of blood analytes of *S. esocinus* and physico-chemical parameters of River Jhelum is given in Table 4, while seasonal variations in hematological parameters of male and female *S. esocinus* are presented in Table 5 and Table 6, respectively. Significant variations were observed in analytes with respect to seasons. Moreover, it was observed that all these values were found within the reference range.

**TABLE 2:** Hematology and serum biochemical reference intervals for snow trout, *Schizothorax esocinus* from River Jhelum. Reference intervals were determined using reference value advisor software following non-parametric method for all measurands (*p* < 0.05) and robust method (*p* > 0.05) for total protein.

Blood analyte	Mean	Median	SD	CV (%)	Min	Max	References interval	90% CI		Anderson darling test, *p*-value
								Lower limit	Upper limit	
Hb (g/L)	94.72	92.85	11.18	11.80	66.2	119.6	78.38–116.35	78.10–78.90	115.42–117.20	<0.01
WBC (×10^9^/L)	14.72	14.50	2.71	18.40	9.00	22.00	10–20	9.50–10.00	19.50–20.50	<0.01
RBC (×10^12^/L)	1.68	1.63	0.25	14.65	1.17	2.34	1.30–2.15	1.28–1.33	2.13–2.19	<0.01
PCV (%)	33.08	32.64	3.32	10.04	26.00	41.1	27.00–39.45	27.00–27.70	39.06–39.87	<0.01
ESR (mm/hr)	1.9	2.0	1.0	53.07	1.0	4.0	1–4	1.00	4.00	<0.01
MCH (pg)	56.77	56.98	3.68	6.48	46.61	66.92	49.62–62.37	48.17–50.14	61.95–63.23	<0.01
MCHC (g/L)	286.49	284.31	17.20	6.00	242.49	349.84	258.91–326.30	254.73–260.74	322.41–334.56	<0.01
MCV (fL)	198.67	199.09	16.39	8.25	161.31	235.25	166.91–226.56	164.83–169.63	224.97–228.01	<0.01
Total protein (g/L)	50.58	50.10	5.95	11.77	26.4	65.9	39.21–61.62	36.95–40.98	61.12–62.82	0.089
Albumin (g/L)	13.29	12.50	3.59	26.62	4.4	27.2	8.20–22.02	8.07—8.50	21.2–22.79	<0.01
Globulin (g/L)	37.28	36.80	5.26	14.12	17.1	54.2	27.58–49.55	26.58–28.92	48.05–49.92	<0.01
Glucose (mmol/L)	5.10	5.01	1.09	21.35	3.00	8.11	3.25–7.18	3.16–3.32	7.05–7.35	<0.01
Urea (mmol/L)	1.55	1.51	0.38	24.31	0.82	2.75	0.96–2.38	0.92–0.99	2.33–2.52	<0.01
Cholesterol (mmol/L)	5.34	5.36	0.92	17.22	3.07	7.02	3.80–6.90	3.50–3.92	6.82–6.92	<0.01

Abbreviations: SD, standard deviation; CV, coefficient of variation; Hb, Hemoglobin; WBC, white blood cell; RBC, red blood cell; PCV, packed cell volume; ESR, erythrocyte sedimentation rate; MCH, mean corpuscular hemoglobin; MCHC, mean corpuscular hemoglobin concentration; MCV, mean corpuscular volume.

**TABLE 3:** Sex based hematological and serum biochemical reference intervals for *Schizothorax esocinus* ^a^ Reference intervals were determined using reference value advisor software following non-parametric method (*p* < 0.05) for all measurands and robust method (*p* > 0.05) for WBC of female and glucose of male.

Blood analyte	Sex	Mean	Median	SD	Min	Max	References interval	90% CI		Anderson darling test, *p*-value
								Lower limit	Upper limit	
Hb (g/L)	M	95.63^a^	94.0	10.83	66.2	118.1	78.84–116.3	76.20–80.20	115.20–117.20	<0.01
	F	93.81^b^	91.8	11.46	75.3	119.6	78.30–116.65	76.3–78.90	115.0–118.50	<0.01
WBC (×10^9^/L)	M	14.41^b^	14.00	2.80	9.00	22.00	9.71–19.50	9.00–10.00	19.50–20.00	0.001
	F	15.03^a^	15.00	2.58	9.5	22.00	10.00–20.29	9.50–10.50	19.50–21.50	0.053
RBC (×10^12^/L)	M	1.70^a^	1.64	0.25	1.27	2.22	1.34–2.16	1.29–1.37	2.13–2.19	<0.01
	F	1.65^b^	1.61	0.24	1.17	2.34	1.27–2.16	1.22–1.32	2.10–2.23	<0.01
Glucose (mmol/L)	M	5.19^a^	5.25	1.06	3.01	8.11	3.17–7.27	3.01–3.27	6.73–8.09	0.059
	F	5.00^b^	4.83	1.11	3.00	7.7	3.29–7.15	3.10–3.38	7.07–7.54	<0.01
Cholesterol (mmol/L)	M	5.23^b^	5.12	0.96	3.09	7.02	3.48–6.93	3.22–3.81	6.80–6.98	0.001
	F	5.45^a^	5.48	0.86	3.07	6.94	3.95–6.88	3.85–4.03	6.78–6.92	<0.01

^a^ The column showing mean values between the sexes with different superscripts are significantly different (Mann Whitney *U* test, *p* < 0.05).

**TABLE 4:** Correlation matrix of blood analytes of *S. esocinus* and physico-chemical parameters from River Jhelum.

	Hb	RBC	WBC	PCV	ESR	MCH	MCHC	MCV	TPR	ALB	GLO	GLC	Urea	CHOL	TEMP	pH	DO	CO_2_	ALK
Hb	1.000																		
RBC	.887^**^	1.000																	
WBC	.200^**^	.207^**^	1.000																
PCV	.863^**^	.820^**^	.193^**^	1.000															
ESR	−.057	−.030	.041	−.022	1.000														
MCH	−.232^**^	−.633^**^	−.091	−.303^**^	−.006	1.000													
MCHC	.496^**^	.359^**^	.062	.031	−.084	.067	1.000												
MCV	−.498^**^	−.731^**^	−.109^*^	−.233^**^	.049	.725^**^	−.608^**^	1.000											
TPR	.248^**^	.263^**^	.143^**^	.212^**^	−.035	−.182^**^	.125^**^	−.219^**^	1.000										
ALB	.175^**^	.197^**^	.073	.164^**^	−.031	−.145^**^	.049	−.145^**^	.537^**^	1.000									
GLO	.145^**^	.153^**^	.084	.141^**^	−.024	−.112^*^	.050	−.116^*^	.765^**^	−.033	1.000								
GLC	.236^**^	.253^**^	.167^**^	.226^**^	−.062	−.168^**^	.080	−.153^**^	.214^**^	.255^**^	.075	1.000							
UREA	.226^**^	.245^**^	.098^*^	.184^**^	−.068	−.159^**^	.119^*^	−.198^**^	.202^**^	.186^**^	.091	.332^**^	1.000						
CHOL	.229^**^	.260^**^	.133^**^	.210^**^	−.061	−.194^**^	.120^*^	−.210^**^	.353^**^	.205^**^	.242^**^	.291^**^	.347^**^	1.000					
TEMP	.453^**^	.439^**^	.227^**^	.393^**^	−.146^**^	−.202^**^	.223^**^	−.287^**^	.370^**^	.328^**^	.220^**^	.405^**^	.400^**^	.471^**^	1.000				
pH	.095^*^	.141^**^	.075	.103^*^	−.012	−.152^**^	−.013	−.105^*^	.052	.110^*^	−.049	.174^**^	.153^**^	.225^**^	.131^**^	1.000			
DO	−.315^**^	−.304^**^	−.150^**^	−.299^**^	−.017	.133^**^	−.135^**^	.163^**^	−.206^**^	−.113^*^	−.157^**^	−.333^**^	−.164^**^	−.251^**^	−.529^**^	−.236^**^	1.000		
CO2	−.186^**^	−.136^**^	−.051	−.159^**^	−.007	−.024	−.089	.020	.203^**^	.093	.239^**^	−.144^**^	−.093	−.205^**^	−.290^**^	−.299^**^	.198^**^	1.000	
ALK	.136^**^	.258^**^	.144^**^	.144^**^	−.098^*^	−.330^**^	.035	−.283^**^	.334^**^	.153^**^	.298^**^	.235^**^	.239^**^	.325^**^	.356^**^	.026	−.064	.198^**^	1.000
** Correlation is significant at the 0.01 level (2-tailed), * Correlation is significant at the 0.05 level (2-tailed)																			

Abbreviations: Hb, Hemoglobin; RBC, red blood cells; WBC, white blood cells; PCV, packed cell volume; ESR, erythrocyte sedimentation rate; MCH, mean corpuscular hemoglobin; MCHC, mean corpuscular hemoglobin concentration; MCV, mean corpuscular volume; TPR, total protein; ALB, albumin; GLO, globulin; GLC, glucose; CHOL, cholesterol; TEMP, temperature; pH hydrogen ion concentration; DO, dissolved oxygen; CO_2_, carbon dioxide; ALK, total alkalinity.

**TABLE 5:** Seasonal variations in hematological and serum biochemical parameters of male *S. esocinus* from River Jhelum^a^.

Parameters	Spring	Summer	Autumn	Winter
Hb (g/L)	93.35 ± 9.12^b^	100.90 ± 10.79^a^	100.01 ± 1.06^a^	88.27 ± 0.75^c^
WBC (×10^9^/L)	14.06 ± 2.99 ^bc^	15.40 ± 2.67^a^	14.96 ± 2.71 ^ab^	13.23 ± 2.42 ^bc^
RBC (×10^12^/L)	1.61 ± 0.20^b^	1.85 ± 0.23^a^	1.81 ± 0.24^a^	1.54 ± 0.15^b^
PCV (%)	32.43 ± 3.03^b^	35.20 ± 3.28^a^	34.47 ± 3.21^a^	31.19 ± 2.54^c^
ESR (mm/hr)	2.04 ± 1.20^a^	1.74 ± 0.87^a^	1.67 ± 0.80^a^	2.00 ± 1.01^a^
MCH (pg)	58.19 ± 3.52^a^	54.64 ± 3.13^b^	55.49 ± 3.28^b^	57.49 ± 3.31^a^
MCHC (g/L)	288.02 ± 11.67^a^	286.77 ± 18.21^a^	290.37 ± 19.12^a^	283.51 ± 18.66^a^
MCV (fL)	202.24 ± 13.21^a^	191.11 ± 13.98^b^	191.90 ± 16.82^b^	203.39 ± 14.45^a^
Total protein (g/L)	46.21 ± 5.10^c^	54.46 ± 5.20^a^	52.87 ± 3.48^a^	49.56 ± 4.65 ^b^
Albumin (g/L)	11.89 ± 3.59^c^	14.82 ± 3.30^a^	13.84 ± 3.79 ^ab^	13.04 ± 3.55 ^bc^
Globulin (g/L)	34.33 ± 2.80^c^	39.41 ± 5.86^a^	39.03 ± 2.41^a^	36.52 ± 3.61 ^b^
Glucose (mmol/L)	4.82 ± 0.76^c^	6.13 ± 0.93^a^	5.40 ± 0.74 ^b^	4.41 ± 0.91 ^d^
Urea (mmol/L)	1.45 ± 0.42 ^b^	1.75 ± 0.34^a^	1.67 ± 0.30^a^	1.33 ± 0.27 ^b^
Cholesterol (mmol/L)	4.79 ± 0.92^c^	6.02 ± 0.71^a^	5.38 ± 0.81 ^b^	4.73 ± 0.81^c^

^**a**^ Values presented as Mean ± SD (*n* = 54 per season). Values with different superscripts within the same row denote significant differences (*p* < 0.05).

**TABLE 6:** Seasonal variations in hematological and serum biochemical parameters of female *S. esocinus* from River Jhelum^a^.

Parameters	Spring	Summer	Autumn	Winter
Hb (g/L)	91.65 ± 10.56^b^	100.52 ± 11.36^a^	97.46 ± 11.27^a^	85.59 ± 5.70^c^
WBC (×10^9^/L)	14.63 ± 2.65 ^b^	15.70 ± 2.84^a^	15.79 ± 2.09^a^	14.02 ± 2.31 ^b^
RBC (×10^12^/L)	1.58 ± 0.22^b^	1.79 ± 0.24^a^	1.73 ± 0.23^a^	1.51 ± 0.16^b^
PCV (%)	32.00 ± 3.07^c^	34.61 ± 3.01^a^	33.23 ± 3.22^b^	31.44 ± 2.67^c^
ESR (mm/hr)	2.02 ± 1.14^ab^	1.87 ± 0.89^ab^	1.70 ± 0.98^b^	2.13 ± 1.05^a^
MCH (pg)	58.12 ± 3.40^a^	56.27 ± 3.07^b^	56.75 ± 3.77^ab^	57.20 ± 4.44^ab^
MCHC (g/L)	286.24 ± 14.97 ^b^	290.17 ± 16.90 ^ab^	293.20 ± 16.77^a^	273.19 ± 12.94^c^
MCV (fL)	203.55 ± 15.49^a^	194.55 ± 15.24^b^	194.03 ± 15.22^b^	208.99 ± 17.63^a^
Total protein (g/L)	44.22 ± 5.45^c^	53.80 ± 4.67^a^	53.21 ± 4.93^a^	50.28 ± 5.07 ^b^
Albumin (g/L)	11.43 ± 3.10 ^b^	14.20 ± 3.13^a^	14.48 ± 3.34^a^	12.43 ± 2.97 ^b^
Globulin (g/L)	32.79 ± 4.26 ^b^	39.60 ± 6.83^a^	38.73 ± 6.26^a^	37.86 ± 3.83^a^
Glucose (mmol/L)	4.71 ± 0.89 ^b^	6.05 ± 1.11^a^	4.94 ± 0.82 ^b^	4.31 ± 0.80^c^
Urea (mmol/L)	1.52 ± 0.36 ^b^	1.79 ± 0.33^a^	1.56 ± 0.43 ^b^	1.35 ± 0.28^c^
Cholesterol (mmol/L)	5.37 ± 0.77^c^	6.02 ± 0.62^a^	5.71 ± 0.79 ^b^	4.68 ± 0.64 ^d^

^**a**^ Values presented as Mean ± SD (*n* = 54 per season). Values with different superscripts within the same row denote significant differences (*p* < 0.05).

## Discussion

Hematological studies reflect the functional status of the fish body (Sidiq and Ahmed, 2020). Among hematological constituents, Hb concentration in fish blood reflects its oxygen supply and the fish strives to uphold the values in as much as steady condition as possible. During the present work, Hb values were significantly higher in male than female fish, similar trend has been observed in *Sander lucioperca* (Zakes et al., 2016)*.* The higher mean hematological values of Hb and RBC observed in male as compared to that of the female could be due to higher physiological activeness as well as metabolic activity in male than the female fish (Sharma et al., 2017). The higher values of hematological parameters in the male are attributed to the action of glycoprotein hormone known as erythropoietin which is known to stimulate erythrocyte synthesis that transport oxygen (Zakes et al., 2016). Since erythropoietin is known to amplify under the influence of the sex hormones, particularly testosterone, this could explain the reason of higher hematological values in male (Golemi et al., 2013). Higher values of Hb, RBC and PCV were noted during summer as compared to other seasons. Similar results have been reported in other freshwater fish species such as *Catla* and *Schizothorax niger* (Pradhan et al. 2012; Sheikh and Ahmed, 2019). The pattern of relatively higher values of RBC and PCV observed in *S. esocinus* during warmer seasons (summer and autumn) as compared to colder seasons was also observed in another fish, *Tinca* and the result was interpreted as an adjustment for increased oxygen intake by the fish body due to the depletion in dissolved oxygen concentration of water bodies during warm months, as rising temperature reduces solubility of oxygen in water bodies (Guijarro et al., 2003; Sharma et al. 2017). Higher levels of WBC in a fish make them proficient to fight infection more effectively than that of others, because of direct correlation between the WBC counts and the immune responses (Douglass and Jane, 2010; Ahmadifar et al., 2019). With respect to sex, female fish had higher WBC values as compared to male. Similar observation of higher WBC in female than the male was recorded in *Heteroclarias* hybrid (Okorie-Kanu and Unakalamba, 2015). Also, the large energy requirements for the growth and maturation of oocytes influence the condition of females and also cause lowered immunity (Kapila et al., 2000; Zakes et al., 2016). This gap could be compensated for by the elevated production of leucocytes in female than in male, as has been previously noted in *Tor putitora* (Kapila et al., 2000). The maximum WBC count was found in the summer and the lowest values were recorded during winter months of the year, similar observations has also been observed in *Cirrhinus mrigala* (Pradhan et al., 2014). While the Mann Whitney *U* test between male and female snow trout showed statistically significant (*p* < 0.05) differences for Hb, WBC, RBC, glucose and cholesterol, the differences in reference intervals are slight but may have some clinical relevance (Table 3). RBC is vital for the transport of oxygen in animals. PCV is useful to check anaemic condition in fishes and is therefore, a reliable guide in aquaculture systems as well as fishery management for assessing fish health from various aspects like nutrition, stress and diseases (Brill et al., 2008; Sharma and Chadha, 2021). The PCV values in fishes usually fall in the range of 20%–45% (Hrubec and Smith, 2000). The observed values of PCV in our study fall within this range suggesting fairly good health of the fishes. Similar values of PCV have also been reported in *Heteroclarias* hybrid (Okorie-Kanu and Unakalamba, 2015). ESR is a common hematological test used as a non-specific measure of inflammation and variations in blood plasma or stress condition may affect ESR level in the fish therefore, this factor can be used as an indicator for fish health (Mozanzadeh et al., 2015). The ESR values in fishes depicted no significant difference between the sexes or seasons. ESR values of current study were similar to those found in *S. davidi* (Zhu et al., 2017). On average, the MCH values in fishes range from 30 to 100 pg, MCV values varies between 150 and 350 fL and MCHC in fishes range from 18 to 30 g/dL (Hrubec and Smith, 2000). The observations of our study do fall within this range. Also, no significant differences were observed in any of the erythrocyte indices with respect to sex. Since the erythrocyte indices are dependent on Hb, RBC and PCV, so the values of erythrocyte indices also varied seasonally. The study of erythrocyte indices in fish is of paramount importance for diagnosing several patho-physiological abnormalities, however, their interpretation demands in-depth information about the multiple number of controlling factors such as age, gender, water quality, capture, handling, and use of anesthetics involved (Clauss et al., 2008).

In addition to hematological parameters, serum biochemical parameters are also used as biomarkers to assess fish health. The plasma or serum protein concentration levels also serve as clinical indicators of health, stress and nutritional condition in the fishes and are often used as a diagnostic tool in evaluating the general physiological status of fish (Zakes et al., 2016). The total protein values are affected by nutritional state, reproductive cycle, habitat and disease or toxins (Coskun et al., 2016). Albumin plays a role in transportation of lipid and general metabolism in fishes while the globulin levels are related to the innate immune response of fish (Andreeva, 1999; Domingo-Roura et al., 2001). The values of total protein, albumin and globulin levels are similar to the values recorded in *C. gariepinus* (Mozanzadeh et al., 2015). In our study, serum total protein, albumin and globulin levels did not exhibit significant variations (*p* < 0.05) with respect to sex. Similar trend was also noted in *Heteroclarias* hybrid (Okorie-Kanu and Unakalamba, 2015). Total protein, globulin and albumin values showed seasonal variations such that higher levels were observed in summer and autumn, while comparatively lower values were recorded in winter and spring seasons. Seasonal variations in these analytes were also noted in *Barilius bendelisis* and *Schizothorax labiatus* (Sharma et al. 2017; Jan and Ahmed, 2020). Glucose is considered as the principal source of energy for the cells of the body. It is transported from the intestines or liver to body cells through bloodstream. Glucose levels are regarded as one of the specific indicators of sympathetic activation during stress conditions and the homeostatic mechanism of the body keeps blood glucose levels within a range (Svoboda et al., 2001; Kulkarni and Bedjargi, 2016). The sex based comparison of serum glucose revealed significantly higher (*p* < 0.05) values in male as compared to female. The plausible explanation of high glucose level in male is due to high deposition of glucose in the cells and ovarian tissues of female that use considerably larger reserves of this monosacchride than do the tissues of the male testes (Zakes et al., 2016). Serum glucose values observed in our study are somewhat similar to the values recorded in a related species *S. niger* (Ruqaya et al., 2012)*.* Seasonally, the glucose content of *S. esocinus* was found highest in summer and lowest values were recorded during winter season, similar trend on serum glucose content has also been reported in *Oncorhynchus mykiss* (Coskun et al,. 2016). Serum urea levels in the present study are similar to those reported in *Clarias gariepinus* (Mozanzadeh et al., 2015). Serum cholesterol is an important steroid metabolite in the cell membranes and transported in the blood plasma of all animals, needed for proper body functions and functions as precursors for the synthesis of sexual hormones (Ruqaya et al., 2012). It serves as the substrate for the synthesis of various important, biologically active compounds, such sex hormones and corticosteroids (Zakes et al., 2016). Moreover, sex based comparison showed that cholesterol levels in female are higher than the values in male. Similar trend was also reported in *S. lucioperca* (Zakes et al., 2016)*.* The results achieved in the present study express the higher reproductive cost of female. In both sexes, the values of urea and cholesterol contents were highest in summer and lowest in winter season of the year. Our findings are conformity with the finding on *C. mrigala* by Pradhan et al. (2014). Among all the serum biochemical parameters significant differences were recorded in case of cholesterol and glucose values between male and female. The variations in hematological and plasma chemistry reference intervals with respect to sex has also been reported in *Alburnus chalcoides* (Moshfegh et al., 2018). Thus, the values observed in *S. esocinus* are consistent with the values found in other freshwater fishes. The current study has implications in terms of addressing the health management of this particular species especially in wild conditions, but its applications in aquaculture are limited due to its currently unexplored culture.

## Conclusion

This work offers a baseline data of hemato-biochemical profiling of *S. esocinus* and how these values fluctuate seasonally. The information may improve the application of clinical chemistry in fish medicine such as health assessment, diagnosis of various subclinical as well as clinical diseases, reference point for future comparative studies, thus enabling better conservation and management strategies, which in turn could assist in rearing this species on commercial basis thereby paving way for better gains to fish farmers and enriching the economy as well.

## Data Availability

The original contribution presented in the study are included in the article/supplementary material, further inquiries can be directed to the corresponding author.
